# Supplementary material for: Haloperidol, Olanzapine, and Risperidone Induce Morphological Changes in an In Vitro Model of Human Hippocampal Neurogenesis
Source: Biomolecules. 2024 Jun 13;14(6):688. doi: 10.3390/biom14060688 (PMC11201986; doi:10.3390/biom14060688)
Supplement: Supplementary file 1 [file biomolecules-14-00688-s001.zip › biomolecules-3006956-supplementary.pdf]

Haloperidol, Olanzapine, and Risperidone Induce Morphological Changes in an In Vitro Model of Human Hippocampal Neurogenesis

Bálint Jezsó [1,2,3,†], Sára Kálmán [4,†], Kiara Gitta Farkas [1], Edit Hathy [1,5], Katalin Vincze [1,5], Dzsenifer Kovács-Schoblocher [1], Julianna Lilienberg [1], Csongor Tordai [1,5], Zsófia Nemoda [5], László Homolya [1], Ágota Apáti [1,*] and János M. Réthelyi [5,*]

Submitted to Biomolecules

Supplementary material

Supplementary Table S1. mRNA expression of dopamine and serotonin receptors in NPCs and 3 weeks old neural cultures. The antipsychotic that acts on the given receptor has been indicated in parentheses. The receptors on which antipsychotics act and are expressed in the in vitro system are highlighted in green. The expressions are indicated in RPKM (Reads Per Kilobase Million).

	RPKM	
**receptors (antipsychotics)**	**NPC_**	**Neu_3_w**
**D1**	**0,172**	**0,247**
**D2 (HP,RP)**	**0,003**	**0,008**
**D3 (HP,RP, OL)**	**0**	**0**
**D4 (HP,RP)**	**0,251**	**1,722**
**D5 (OL)**	**0**	**0**
**5 HT 1A, G protein-coupled**	**0**	**0**
**5 HT 1B, G protein-coupled**	**0**	**0,232**
**5 HT 1D, G protein-coupled**	**0,067**	**0,144**
**5 HT 1E, G protein-coupled**	**0,001**	**0,038**
**5 HT 1F, G protein-coupled**	**0**	**0**
**5 HT 2A, G protein-coupled (RP,OL)**	**0,013**	**0,026**
**5 HT 2B, G protein-coupled (OL)**	**0**	**0**
**5 HT 2C, G protein-coupled (OL)**	**0,011**	**0,077**
**5 HT 3A, ionotropic**	**0**	**0**
**5 HT 3B, ionotropic**	**0,002**	**0,008**
**5 HT 3C, ionotropic**	**0**	**0,006**
**5 HT 3D, ionotropic**	**0**	**0**
**5 HT 3E, ionotropic**	**0**	**0**
**5 HT 4, G protein-coupled**	**0**	**0**
**5 HT 5A, G protein-coupled**	**0**	**0**
**5 HT 6, G protein-coupled (OL)**	**0**	**0,003**
**5 HT 7, adenylate cyclase-coupled (RP)**	**0**	**0,002**

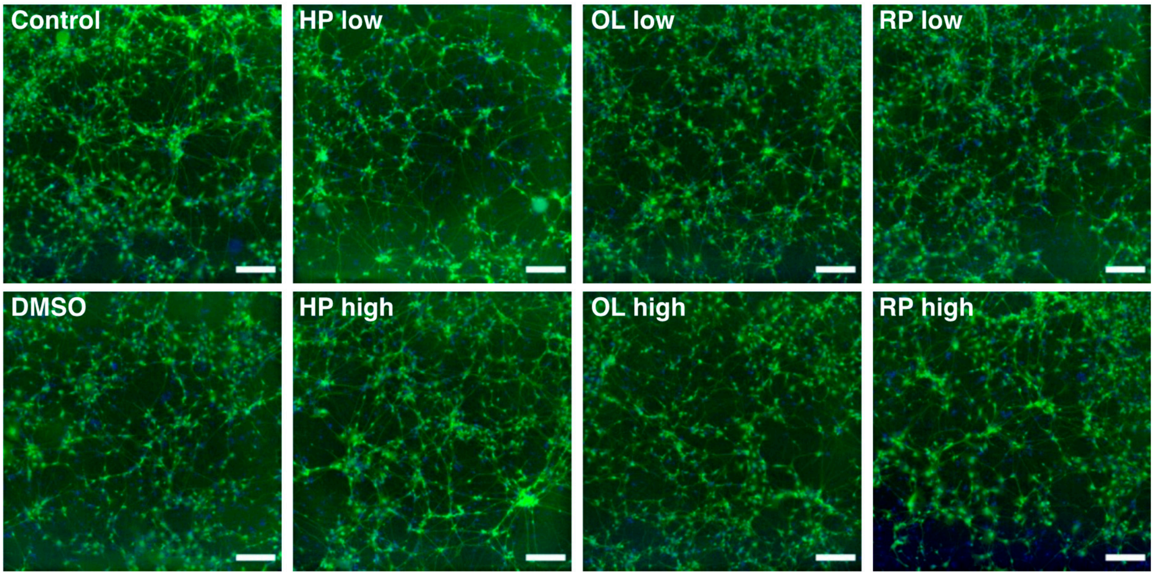

Supplementary Figure S1. Representative pictures of neurite outgrowth measurements. Scale bars represent 200 µm.

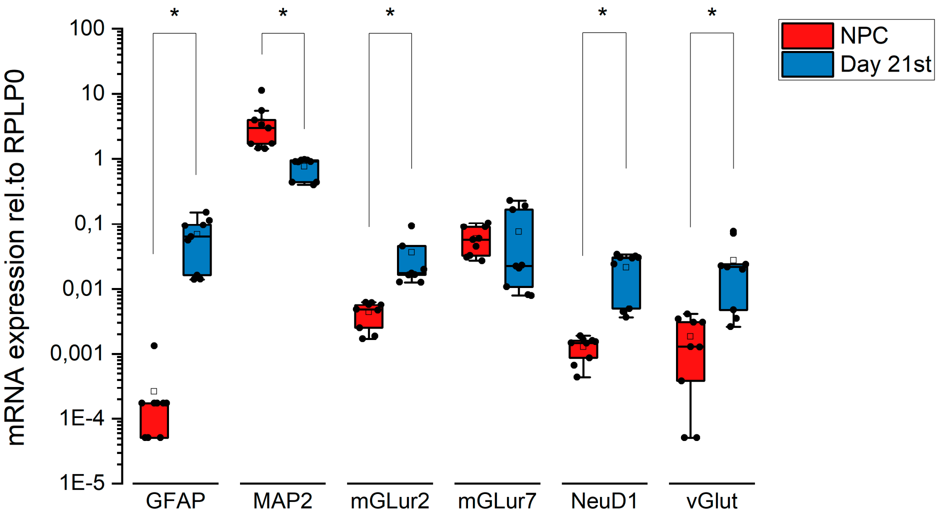

Supplementary Figure S2. mRNA expression profile of NPC and neural cells. Quantification of mRNA expression of selected markers which are essential for lineage specification in NPCs, and 21 day old differentiated neural cultures. The increase in the expression levels over time shows that the differentiation is progressing in the cultures. During statistical analysis Kruskal-Wallis test and post hoc Dunn's test were done.

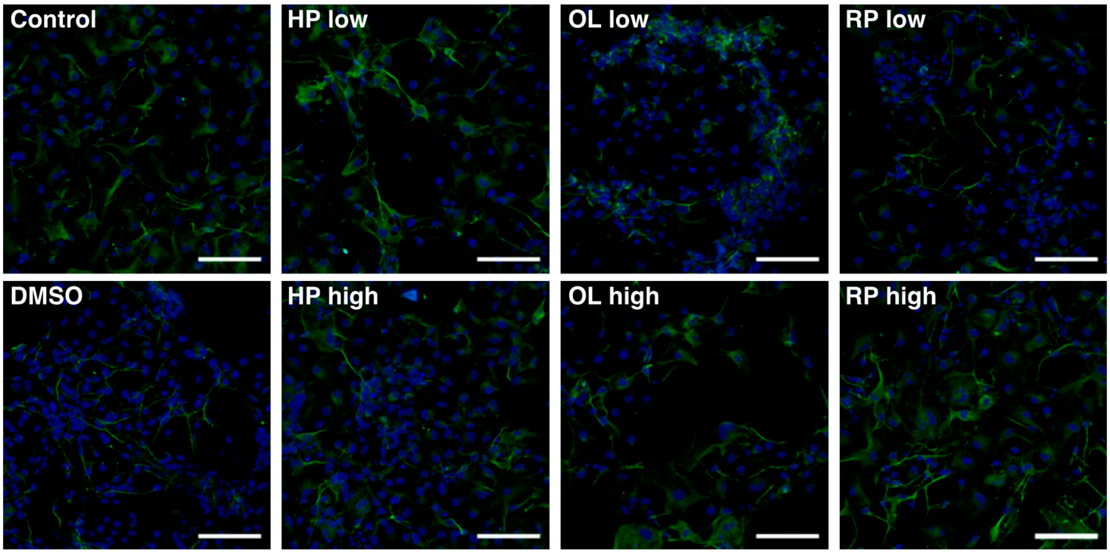

Supplementary Figure S3. Representative pictures of MAP2 staining. The MAP2 protein shows uniform, easily detectable staining in 3-week-old differentiated neurons. Scale bars 100µm.

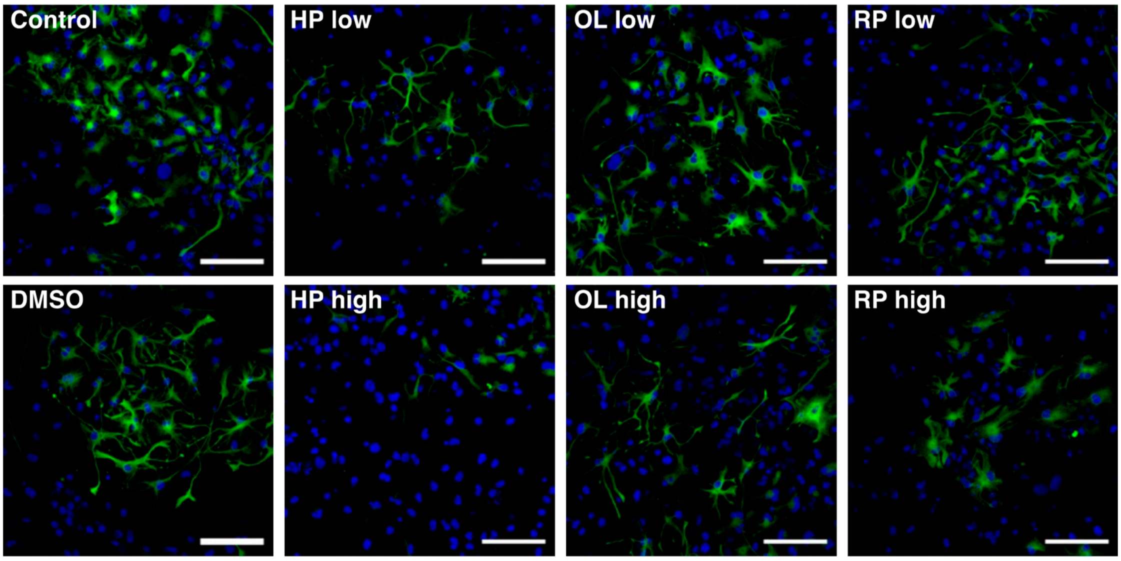

Supplementary Figure S4. Representative pictures of GFAP staining. The GFAP protein shows well-detectable staining in the 3-week-old differentiating cultures, but only in the case of some cell groups. Scale bars 100µm.

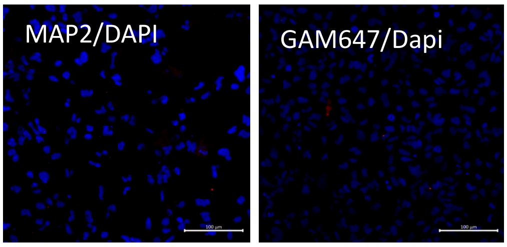

Supplementary Figure S5. Representative pictures of MAP2 staining in NPCs. MAP2 protein is below the detection limit measured with the same antibody that shows significant staining in neurons. No staining is also visible in the control stained only with secondary antibody. Scale bars 100µm.
